# London Hospitals of To-Day

**Published:** 1911-04-15

**Authors:** 


### LONDON HOSPITALS OF TO-DAY.

### I. THE PRINCE OF WALES' GENERAL HOSPITAL, TOTTENHAM.

Tottenham, now a vast district with five surrounding suburbs, is the creation of the last twenty years, and that perhaps explains why the Prince of Wales' General Hospital is still unrecognised by the general public as a great institution, although it has 125 beds and serves a district the population of which increases at the alarming rate of ten thousand persons a year. This population consists largely of men and women who only sleep in the district, and who spend their days at work in the great stores of the West End or in the factories of the City. This is the material of which the hospital population is made: in other words, it is one more in need of treatment than able to secure it for itself. To this type of shop assistant, clerk, and mechanic is now added a new flux of labour, which is following the factories that the local advantages of the railway and canal are yearly causing to be built. It is astounding to think that this multitudinous district was open country within the memory of men now in their prime, and its sudden uprising has produced an immediate hospital problem that was foreseen by no one. Many hospitals grow with their district; Tottenham was densely populated before it had a hospital at all, in the modern sense. The Prince of Wales' Hospital, as we see it to-day, dates virtually from 1898, when a complete reorganisation of the foundation out of which it has grown took place in the first year of Sir Francis Cory-Wright's chairmanship.

Such, then, in the merest outline, is the district which the Prince of Wales General Hospital has to serve. How has the hospital attempted to deal with it? In 1906 and 1907 one wing of the hospital was extended, a new theatre was built, and rooms for the resident medical and nursing staffs were added. This was the first step in a necessary policy of extension. The hospital has never been abreast of the work demanded of it, or, as the words of this year's report put it, "only about one out of every five applications for admission can be granted." It is clear, then, that further building is necessary. Fortunately the hospital possesses a site which is capable of very extensive development, a site sufficiently large more than to double the present size of the building without affecting the open spaces which now form such a useful attraction to the place. In 1907, as stated above, one wing of the hospital was extended, and, the building being symmetrical in shape, it is now hoped to extend the other wing in the same way. This addition would add twenty-four beds to the hospital if carried out. But it must not be supposed that this is all which is required. The new factories of which mention has been made have added to the number of accidents which occur in the district, and this fact at once draws our attention to the casualty and out-patient department.

It is here that the most uncritical observer would say at once that reconstruction was most necessary. These buildings have not been touched since they were built, at a time, in other words, when there was three times less work to do than there is to-day. The out-patient hall, with its old-fashioned plank floor, is quite inadequate for the reception of the three hundred or more persons who congregate there every day. Only by a series of contrivances can they be dealt with at all. There are no observation beds, there is no isolation room, and the corridors are narrow passages, like those of a private house, in which it is impossible to prevent crowding. Immediately over the out-patient hall is the children's ward; a contiguity that sums up in itself the unsatisfactory state of the present arrangements and gives to anyone an idea of the period from which these buildings date. The science of hospital construction has grown so rapidly of recent years that perhaps the out-patient department at the Prince of Wales' Hospital seems more ancient than it really is, but of the reality of that growth no better example could be given than a comparison of these arrangements with any typically up-to-date unit, like that just opened at Liverpool, or at King's College, or again at Great Ormond Street. A standard of efficiency in hospital construction, and in administration, too, though perhaps less completely, has been created, and it is this which makes the remodelling of the out-patient department essential at Tottenham. Moreover, the *x*-ray and electrical departments, which, it should be added, have achieved some exceptionally good results, are housed in what can be called only temporary quarters, for carpentry has been called in to make shift with the rooms which they occupy. The pathological department and mortuary are in the same primitive condition, being simply outhouses converted to their present purpose.

With such a state of affairs the Board can hope for nothing but reconstruction, and plans have been prepared by Mr. Rowland Plumbe which embody suggestions for new and up-to-date buildings. These plans, briefly, suggest the demolition of the present out-patient hall altogether, and the building in its place of a block which shall contain the casualty department on the ground floor, and above that two wards, over one another, on the top of which will be some nurses' bedrooms. Thus the total number of beds added by the wing extension and these wards would be forty. Behind this block, again, would be the new out-patient department, and, if the scheme were completed, a pathological department and mortuary would arise on the site of the present adaptations. Such a scheme would cost £25,000 in capital, and £2,500 a year to maintain.

That is a large sum of money, but it must be found if the hospital is to continue the struggle against the yearly-growing demands of this district, for somehow or other, in spite of every difficulty, the work has been done. The last two years have shown a clean balance-sheet, and the sum on the right side which was over in each case has helped to reduce the hospital's debt, which now stands, we believe, at about £6,000. As regards the money for the extension scheme, two methods of raising it suggest themselves: an appeal to the locality, and a reminder to the owners of the great businesses above referred to that the health of their employés is dependent on the Prince of Wales' Hospital. The appeal which would be made in Tottenham has this great encouragement—that the district has decided that the local memorial to King Edward shall be in connection with the hospital; and if this decision be acted upon it is highly probable that the surrounding districts of Wood Green, Edmonton, and Walthamstow, will associate themselves with it. This, too, is Coronation year, and there can be no doubt that money subscribed to the hospital would be the most advantageous way of celebrating it without waste and with permanent benefit for the people. Tottenham has a great opportunity to acknowledge the ten years of effort which the hospital has made on its behalf. That struggle has been only outlined here, and plans would be necessary clearly to indicate the proposed improvements in detail. A great hospital is in the making and deserves the utmost support in this critical moment of its history.

